# Solitary Pulmonary Hematoma Radiographically Indistinguishable from Mediastinal Tumor

**DOI:** 10.1155/2020/8821137

**Published:** 2020-12-24

**Authors:** Teruya Komatsu, Naoki Date, Takuji Fujinaga, Akira Hara, Tatsuo Kato

**Affiliations:** ^1^Department of General Thoracic Surgery, National Hospital Organization Nagara Medical Center, Gifu, Japan; ^2^Department of Tumor Pathology, Gifu University Graduate School of Medicine, Japan; ^3^Department of Respiratory Medicine, National Hospital Organization Nagara Medical Center, Gifu, Japan

## Abstract

Solitary pulmonary hematoma is a rare consequence of blunt chest trauma. Moreover, there has been no reported case of solitary pulmonary hematoma radiographically diagnosed as a posterior mediastinal tumor. We present the case of a 63-year-old man who was referred for an oval-shaped opacity at the left paraspinal area on a chest X-ray. Chest computed tomography showed a well-circumscribed posterior mediastinal tumor on the left paraspinal lesion with extrapleural sign and callus formation on the left ribs posteriorly (7^th^ to 11^th^ ribs). The tumor was thoracoscopically confirmed to be a subpleural pulmonary tumor of the left lower lobe, and wedge resection was performed. Histological examination confirmed the diagnosis of pulmonary hematoma. On reviewing the callus formation of the ribs, which was suggestive of rib fractures, the pulmonary hematoma was determined to be traumatic in origin. The postoperative course was uneventful. We reviewed a rare case of pathologically proven traumatic solitary pulmonary hematoma. The rarity of this case is enhanced because the hematoma initially appeared to be a posterior mediastinal tumor.

## 1. Introduction

Pulmonary hematoma refers to the presence of blood within the alveolar and interstitial spaces and is most often caused by blunt thoracic trauma. It has also been reported as a pulmonary pseudocyst and is most commonly found in children and young adults [1, 2]. Blunt chest trauma rarely leads to pulmonary hematoma, and a history of trauma and chest computed tomography (CT) findings are usually sufficient to confirm its diagnosis [3].

We report a case of traumatic solitary pulmonary hematoma (SPH) for two reasons; firstly, our pulmonary hematoma was radiographically indistinguishable from a posterior mediastinal tumor, and secondly, pathologically proven traumatic SPH has rarely been reported in the literature.

## 2. Case Presentation

**Figure 1:**
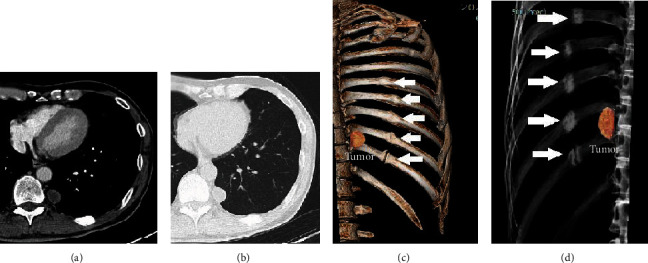
(a) Axial CT enhanced with intravenous contrast, mediastinal window. (b) Axial CT, pulmonary window. (c, d) Three-dimensional reconstruction using the CT of the chest (right ribs removed). Paraspinal oval mass in the lower thoracic spine measuring 20 mm in diameter. The capsule enhances slightly with contrast. The tumor is well-circumscribed without intraspinal extension. The callus formations in the posterior ribs (7th to 11th) can be observed, suggesting previous rib fractures (c, d; arrowheads).

**Figure 2:**
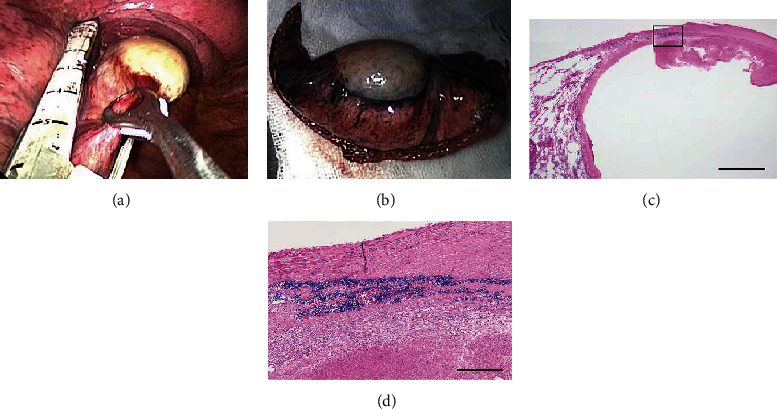
(a) Intraoperative picture of the subpleural mass, with stapler applied. (b) Wedged-out section of the left lower lobe, showing the subpleural mass. (c) Microphotographs of sections stained with Victoria blue and HE stain. (d) High-power field of the rectangle in (c). The elastic layer of the visceral pleura is stained in blue. The hematoma was located inside the visceral pleura, indicating that the lesion is intrapulmonary. Scale bars in (c, d) are 2 mm and 250 *μ*m, respectively.

The patient was a 63-year-old man with a history of a minor fall and subsequent traumatic subdural hematoma 3 months prior. He had no history of anticoagulant use and had no significant family history. He had quit smoking 23 years before. The patient was referred to us for the investigation of an abnormal shadow detected by chest X-ray during an annual health screening. Chest computed tomography (CT) revealed a paraspinal 20 mm diameter oval mass abutting the lower thoracic spine. The tumor showed extrapleural sign and was well-circumscribed without intraspinal extension. The capsule of the mass was slightly enhanced with intravenous contrast (Figures 1(a) and 1(b)). Close to the tumor, callus formations were observed on the 7th through 11th ribs posteriorly, suggestive of previous nondisplaced rib fractures (Figures 1(c) and 1(d)). Hematological examination did not yield any significant findings. Under a preoperative diagnosis of mediastinal tumor, video-assisted thoracoscopic surgery was performed. Unexpectedly, the tumor was visualized to be subpleural within the left lower lobe. The tumor was resected using wedge resection of the left lower lobe and pathologically diagnosed as SPH (Figure 2). Considering the several rib fractures adjacent to the tumor, the hematoma was likely to have been caused by previous chest trauma. The postoperative course was uneventful. The follow-up was done for one month after discharge and was discontinued because of the benign nature of the hematoma.

## 3. Discussion

Nonpenetrating chest trauma often causes pulmonary contusion and intrapulmonary hemorrhage. Pulmonary hematoma, which is caused by laceration of the visceral pleura during lung injuries following blunt chest trauma, is a much less frequent sequela than pulmonary contusion and intrapulmonary hemorrhage [1]. Cases of multiple lesions are rare, and those of solitary lesions are rarer [4]. Previous reports have revealed that the incidence of posttraumatic pulmonary hematoma has been 1–3% after that of nonpenetrating chest traumas in adults, and that it is often seen in children or young adults [5–7].

Radiographical diagnosis in the present case was challenging. Firstly, the sharply marginated spherical mass adjacent to the paravertebral space on the CT scan with the extrapleural sign (Figure 1(b)) strongly suggested that the tumor had a chest wall origin. This indicated that the tumor was likely to be a posterior mediastinal tumor that was either neurogenic or a nerve sheath tumor [8, 9]. Secondly, the mass did not contain an air-fluid cavity, which is a characteristic radiographic sign for pulmonary hematoma [10]. In the present case, the patient did not recognize his chest trauma, which was probably caused by the previous minor fall. This may have complicated the differential diagnosis.

Treatment usually involves conservative therapy, as hematomas are self-resolving [11]. According to previous reports, the mean time for resolution of blood-filled traumatic pulmonary pseudocysts, which were identical to the present case, was 77.1 to 145.8 days [12, 13]. Clinical courses are mostly benign; however, close follow-up is recommended because of some rare but serious complications such as hemopneumothorax or cyst infection [1].

Surgical resection is not commonly performed owing to the self-resolving nature of the cyst [14]. Therefore, reports of pathologically diagnosed pulmonary hematomas are scarce. A previous report revealed that 3 of the 25 patients with pulmonary hematoma underwent resection and that microscopic examination of the pulmonary hematoma showed numerous hemosiderin-laden macrophages and fibrosis in the surrounding tissue [5].

## 4. Conclusion

SPH is an uncommon complication of blunt chest trauma. This is the first report of pathologically proven SPH which was radiographically indistinguishable from a posterior mediastinal tumor.

## Data Availability

The data used to support the findings of this case report are included within the article.
